# Jewish Values in Medical Decision-making for Unrepresented Patients: A Ritualized Approach

**DOI:** 10.5041/RMMJ.10441

**Published:** 2021-07-20

**Authors:** Jason Weiner

**Affiliations:** Senior Rabbi & Director, Spiritual Care Department, Cedars-Sinai, Los Angeles, CA, USA

**Keywords:** Clinical ethics consultation, medical decision-making, unbefriended patients, unrepresented patients

## Abstract

Determining appropriate care for patients who cannot speak for themselves is one of the most challenging issues in contemporary healthcare and medical decision-making. While there has been much discussion relating to patients who left some sort of instructions, such as an advance directive, or have someone to speak on their behalf, less has been written on caring for patients who have nobody at all available to speak for them. It is thus crucial to develop clear and rigorous guidelines to properly care for these patients. The Jewish tradition offers an important perspective on caring for unrepresented patients and determining approaches to guide care providers. This article develops an understanding of fundamental Jewish principles that can provide clear guidance in navigating this challenge. It applies those values to a specific set of suggested behaviors, one of which adds a novel ritualized component to what has been recommended by bioethicists in the past.

## BACKGROUND

One of the most challenging issues in contemporary healthcare and medical decision-making is how to care for patients who cannot speak for themselves. While there has been much discussion related to proper care for patients who left instructions or have someone to speak on their behalf, less has been written on caring for patients who have nobody available to speak for them.^1^ It is thus crucial to develop clear and rigorous guidelines to properly care for these patients. As we seek to develop approaches to guide care providers, the Jewish tradition offers an important perspective for this discussion, although very little has currently been written on it from a Jewish viewpoint.

This article presents an understanding of some fundamental Jewish principles that can provide clear guidance in navigating this challenge. I then apply those values to a specific set of suggested behaviors, one of which adds an additional original component to what has been recommended by bioethicists in the past. The aim of these suggestions is not to impose Jewish values on patients, but to use the Jewish tradition to help develop a new approach to a very complex and challenging area of healthcare. This is not to suggest that any bioethicists or clinicians should adopt Jewish values, but that some of these ideas, which are informed by Jewish perspectives, may benefit those thinking about how to best approach these complex issues in a way that might enhance their clinical care. It may be especially meaningful to Jewish practitioners or anyone working with patients.

## MEDICAL DECISION-MAKING FOR UNREPRESENTED PATIENTS

When there is no one available who is legally recognized as able to speak on behalf of an incapacitated patient, the process of making important healthcare decisions on their behalf is especially difficult. Making appropriate decisions for them can be excruciating, especially when virtually nothing is known about them as an individual, sometimes not even their names (an “unidentified patient”), as frequently occurs with individuals experiencing homelessness, for example. Many American hospitals care for an alarmingly high number of these patients. They are often referred to as “adult orphans,” or “unbefriended,” “isolated,” or “incapacitated patients without advocates,”^2^,^3^ but the most common term is “unrepresented.”^4^ Such patients currently account for over 5% of deaths in intensive care units, and the numbers are increasing, particularly amongst the elderly, homeless, and mentally disabled.^5^ The situation became even worse during the COVID-19 pandemic due to patients’ confusion and isolation as a result of strict visitation policies, causing significant moral distress to clinicians.^6^

These patients are some of the most vulnerable people in our society, and since so little is known about them as individuals, making medical decisions for them is one of the most difficult and controversial challenges that arises in hospitals and bioethics today.^2^ As a result, they are often exposed to either overtreatment, under-treatment, or delayed treatment, and may often receive medical care that conflicts with their own preferences, values, and best interests.^7^ There is no uniform decision-making standard to guide care providers in these cases, nor is there consensus on the proper procedures, and there are very few laws or policies in place to protect this population.^2^

Applying the “best interests standard” can be challenging, because it is often not clear which decision is actually in a given patient’s best interest. Therefore, it is ideal to strive for “substituted judgment” (which means the decision maker must attempt to determine what *the patient would have wanted* if they were competent) to the extent possible, even though that is not always clear either. However, what often happens is simply that an individual physician unilaterally makes all healthcare decisions, with almost no oversight.^8^ This situation is problematic, because giving one person so much authority risks treatment plans that are not carefully thought out or are made based on a conflict of interest, such as institutional financial pressures.^2^ Furthermore, studies show that physicians often simply make decisions based on their own preferences, not the patient’s values.^2^ This result may come about because physicians have not had the opportunity or taken the time to get to know the patient in depth, leading to possible negative assumptions, mistreatment, or treatment that is discordant with the patient’s actual wishes. In addition to that, because physicians often rotate and each one may have different views about proper care plans, unrepresented patients may be exposed to a lack of continuity of care and further arbitrariness in treatment decisions.^4^ Moral guidance is needed to support these patients and their healthcare providers, and although traditional Jewish law does not afford unlimited decision-making autonomy to patients, their own goals and preferences can often be relevant in determining appropriate interventions.

## JEWISH VALUES AND LAWS

Two of the most fundamental and important Jewish values may provide us with significant guidance on this issue. The Talmud teaches that Rabbi Akiva regarded “Love your neighbor as yourself”^9^ as the single greatest encompassing principle of the Torah.

Another of the great sages, Ben Azzai, responded, that the verse “This is the book of the generations of Adam,”^10^ is an even more all-encompassing principle than that, because this verse broadens the requirement to all of humanity, since everyone is created in the image of God.^11^ When fleshed out and applied to bioethics, these core values can have a deeper meaning in healthcare by demonstrating that, more than respecting autonomy, a primary focus of interactions with patients should be the inherent duties that healthcare providers have to care for every individual in their care, as will be explained below.

### Love for Fellow People

The “golden rule” of “Love your neighbor as yourself” adds a level of personal responsibility to the bioethical ideal of substituted judgment and can serve as an important anchor for it. In addition to Rabbi Akiva referring to this commandment as the major encompassing principle of the Torah, the Talmud records that the great sage Hillel referred to it as “the entire Torah; the rest is just commentary,” as he rephrased that directive into: “That which is hateful to you, do not do to your neighbor.”^12^ This teaching thus demands both seeking to benefit others and trying to avoid causing them harm. It is seen as a foundation for much of Jewish law relating to medical interventions, such as the requirements to attempt to heal the sick,^13^,^14^ visit the sick, comfort mourners, escort the dead,^15^ and avoid infecting others.^16^ It similarly bestows permission to utilize palliative care^17^ and prohibits the desecration of a corpse,^18^ among many more such examples.

Seeing this “golden rule” simply as treating others the way *you yourself* would want to be treated has led some bioethicists to critique its usefulness in clinical practice because studies have shown that when healthcare providers attempt to infer their patient’s beliefs and desires from what they assume their own preferences would be under similar circumstances, they often make mistakes in predicting a patient’s wishes or beliefs.^19^ However, many classic commentaries understand this verse as a Biblical obligation to *show love* for others in the way that you would want *if you were in the other person’s circumstances*.^20^ Some even say that this commandment is about respecting other people’s autonomy by *acting* lovingly toward them in the way that you wish they would *act* lovingly toward you.^21^ Everyone is different and has different needs. It is thus reasonable to assume that most people want to be treated in accordance with their own goals, values, and preferences, as much as possible. This school of thought sees the “golden rule” as attempting to understand another person’s own narrative, experience, beliefs, and desires. Accordingly, this verse indeed commands us to treat others not “as you would have them do unto you,” but as *they* would have you do unto them (assuming it is not an act that violates Jewish law).

Indeed, this value leads to a profound ideal in the Mishnah, which states that one of the ways Torah is acquired is by “sharing the burden of others” (“*nosei be’ol im chavero*”).^22^ Some of the leading thinkers of the Jewish ethics and character development movement (*mussar*) explain that this means that one must strive to the utmost to understand and feel another person’s situation from within that person’s own context and life experience. This commandment thus supports the need to strive to provide substituted judgment to the greatest extent possible when making decisions on behalf of unrepresented patients (see suggestion 1 below).

### Divine Image

The first ethical teaching of the Torah is the theological claim that all humans are created in the image of God.^23^ This teaching imbues human beings with responsibilities^24^ and is the basis for many Torah commandments, including the prohibition against murder,^25^ the obligation to bury the dead,^26^ and the duty to save life.^27^ Furthermore, this value leads to the category of Jewish law known as “*kevod habriyot*,” human dignity,^28^ which requires treating all people with basic respect and dignity. This value is so crucial that concerns for protecting human dignity override much of Jewish law, particularly in order to avoid embarrassing people and preserving their reputation.^29^–^32^ Although people can act in ways that betray the dignity of their image of God, or can be treated in ways that are an affront to their image of God,^33^ a person never loses their inherent image of God, even if the person is incapacitated and, indeed, even after death.^34^

Based on this understanding, the Talmudic sages created a profound ritual that is especially relevant for our discussion of caring for unrepresented patients. The Torah has certain categories of prohibitions that incur the death penalty. However, the rabbis severely limited and restricted the practical application of capital punishment. One of the ways they did so was by means of very careful examination of the witnesses in a capital case. Before giving potentially incriminating testimony, the witnesses had to be told a number of things by the court, including, “Adam was created alone to teach you that anyone who destroys one soul, the verse blames them as if they destroyed an entire world, but anyone who sustains one soul, the verse credits them as if they sustained an entire world.”^35^ They would then go on to tell the witnesses that “this was done due to the importance of maintaining peace among people, so that one person cannot say to another: My progenitor is greater than yours … it also tells of the greatness of God, since when a person stamps several coins with one seal, they are all similar to each other, but the supreme King of kings, the Holy Blessed One, stamped all people with the seal of Adam, the first person, yet not one of them is like another. Therefore, every person is obligated to say: ‘The world was created for me.’”^36^

This ritual reflects the view that for a witness to be relied upon in life-and-death matters, they must be reminded of the tremendous import and fundamental dignity of all human life, created in the image of God. This statement can be summarized as declaring three things^37^:

1. that every human life is of immeasurable value;
2. that every human life is of equal value;
3. that everyone is unique.

This Talmudic ritual, taken to remind people of the human dignity inherent in every person, but especially those most vulnerable, can serve as a model to be applied in contemporary care for unrepresented patients. Similar to those accused of crimes, whose fates are determined by a committee, every patient, no matter their condition, deserves the utmost respect and equitable treatment in accordance with their own individual values to the extent that is possible.

## DECISION-MAKING

### Striving to Learn About Patients

These values may offer valuable guidance for how to approach making decisions for unrepresented patients. In order to respect the dignity and uniqueness of each person, it should not be assumed that, just because a patient is unrepresented, they do not have values and preferences. Most likely someone, somewhere, knows something about them,^38^ and so whenever possible, before making a decision, there should be a diligent search to attempt to find a surrogate, or expand the list of those who can be considered a valid surrogate, in order to increase the chances of finding a person who has information about this individual’s goals, values, or preferences.^2^ However, it is often very difficult to locate such an individual, and at times there truly is nobody who knows a given patient.^2^ Yet, even then, it may be possible to find some sort of evidence about how an individual lived their life in order to attempt to infer some of their values.^3^ These Jewish principles suggest that not only is this an expectation of some contemporary bioethicists, but that there may also be a Biblical *obligation* to make every attempt to do so.

### Diverse Interdisciplinary Decision-making Committees

Beyond that, and particularly when nothing at all can be learned about a patient or anyone whom they might know, respect for the inherent value and dignity of each human being, as well as the equality of all persons, demands that hospitals develop rigorous decision-making processes for these patients in order to ensure that they are treated fairly and with dignity—not just out of respect for their autonomy, but because there is an *obligation* to care for individuals this way.

Some states in the USA authorize clinicians to make the decision with almost no oversight,^3^ and others require the safeguard of a second physician or committee to oversee medical decisions made on behalf of unrepresented patients.^2^ Yet other American states have a tiered approach, in which they allow an attending physician to make routine decisions alone, but require approval from another physician for more risky, major medical treatments, and they require consultation with an independent physician or multidisciplinary committee (or court approval) for decisions involving life-sustaining treatment.^2^ Although it is essential to ensure a decision-making process that is accessible, quick, convenient, and cost-effective, utilizing the values outlined above for cases which are neither urgent nor routine would seem to require engaging in the most rigorous safeguards of expertise, neutrality, and careful deliberation.^4^ I therefore believe that in decision-making for unrepresented patients, Jewish ethics would advocate for following the more demanding process of involving a diverse interdisciplinary committee, comprising not only the treating clinicians, but also individuals representing that patient’s own religious or cultural community, whenever necessary and possible.

Indeed, in addition to the careful oversight of witnesses in capital cases in a Jewish court, the sages of the Talmud created the counterintuitive policy that if all twenty-three judges deciding on a capital case vote unanimously to convict, then the defendant goes free,^39^,^40^ because complete unanimity indicates that not enough of an attempt was made to explore and understand different arguments and perspectives.^41^ Utilizing an interdisciplinary committee to carefully deliberate would thus reflect Jewish values, in that it would seek to avoid bias and conflict of interest, and to safeguard procedural fairness, transparency, consistency, and oversight, while ensuring that multiple, carefully weighed perspectives are incorporated. This process should thus be utilized for complex cases even when state laws permit a far simpler standard, because it offers a higher likelihood of fair and rigorous decision-making than does a single person making unilateral decisions without oversight. Achieving good ethical consensus is not merely about agreement, but about who is agreeing and the quality of the deliberative process.^42^

### Ritualizing These Values

The values detailed above encourage following the strictest standards of the bioethicists quoted in this article^2^ and utilizing an interdisciplinary committee rather than simply having an individual physician unilaterally make all healthcare decisions. However, I believe these values require us to go even further than what has been previously recommended and to take steps to ritualize these ideals, based on the formal statement about the image of God read to witnesses in capital cases, mentioned above. People’s busy schedules and the high volume of these sorts of cases may unfortunately lead to some practitioners occasionally forgetting that an unidentified patient is more than just a body lying in a hospital bed. Even when a practitioner values something, research has shown that an act of “priming,” which is simply reading a statement or being reminded of one’s values prior to being asked to engage in an act, increases the likelihood of compliance with one’s own values and keeping their positive intentions in mind.^43^,^44^ I therefore recommend that prior to meeting to make medical decisions on behalf of unrepresented patients, a brief formal statement should be read, reminding each participant of the value, equality, and uniqueness of every human being, modeled after the Talmudic statement made to witnesses in capital cases, in order to protect highly vulnerable populations. Ideally this statement should include the patient’s name, some known detail about them, or display a photo of them, if possible. This statement should be as inclusive as possible and refer to the extent of the healthcare provider’s duty to care for others and to provide care that is as concordant with the patient’s own goals and values as possible, highlighting the dignity of each person and the magnitude of the decisions being made on their behalf.

In the diverse healthcare environment, this statement could be something as simple as reading aloud an inclusive and non-sectarian line such as, “Before engaging in making decisions on behalf of this patient [insert name if known], we hereby recognize our patient’s inherent value and uniqueness and commit ourselves to striving to understand who this patient is, to fulfill our duties toward and care for this patient equitably and with dignity, to the best of our ability.”

In the Jewish tradition, ritual practices, such as the Passover *seder*, are frequently utilized to help transform abstract ideals into living practices that shape character.^45^ Similar types of priming statements are made before performing many mitzvot. For example, some traditional Jewish prayer books suggest beginning one’s day by proclaiming, “I hereby take upon myself to fulfill the commandment to ‘love your fellow person as yourself.’” Indeed, the idea of the physician’s oath has been common in the history of medicine, and many have suggested Jewish versions to be recited by doctors prior to engaging in medicine^46^ and by patients prior to receiving treatment.^47^

Likewise, the idea of healthcare providers engaging in helpful rituals is not unheard of in contemporary healthcare. For example, many emergency rooms and ICU’s have implemented “post-code pauses” (also known as “post-resuscitation debriefings”) in which, following a resuscitation, trauma, or death, staff engage in a formalized moment of silence, followed by some simple reflections, questions, and debriefing in order to pay homage to the patient and process their own thoughts and feelings before continuing their shift. These pauses have been shown to help healthcare providers feel more present and able to meet the needs of all of their patients.^48^,^49^

## CONCLUSIONS

Although Jewish ethics come to many of the same conclusions as some of the most rigorous standards put forward in secular bioethics, as quoted in this article, these conclusions come from different starting points, which add additional insights and responsibilities. Most bioethicists who write on the topic of making decisions for incapacitated patients base their models primarily on respect for the patient’s autonomy.^50^,^51^ This Jewish approach, on the other hand, focuses less on patients’ *rights*, and more on care providers’ *obligations* to care for them and to protect their intrinsic dignity, which can have a significant impact on how and why decisions are made.^52^ After all, not only does being created in the image of God confer human dignity, it also means that our lives belong to God, and thus that our own autonomy is not the primary value.^43^ This duty-based perspective requires a very high threshold of striving to ensure that the right thing be done and in the right way. Basing such decisions on these universal Biblical values and ideology can serve to heighten care providers’ sensitivity to treating each patient with dignity and their sense of obligation to do so. Moreover, ritualizing this process and verbalizing these values adds an additional reminder that can help ensure that they are in fact acted upon on a regular basis.
